# Efficacy of Second-Line Lenvatinib After Atezolizumab–Bevacizumab and Durvalumab–Tremelimumab in Unresectable Hepatocellular Carcinoma: Association with Prior Immunotherapy Response

**DOI:** 10.3390/cancers18132095

**Published:** 2026-06-28

**Authors:** Teiji Kuzuya, Hisanori Muto, Yoshihiko Tachi, Gakushi Komura, Takuji Nakano, Hiroyuki Tanaka, Kazunori Nakaoka, Kohei Funasaka, Mitsuo Nagasaka, Ryoji Miyahara, Eizaburo Ohno

**Affiliations:** 1 Department of Gastroenterology and Hepatology, Fujita Health University, Toyoake 470-1192, Aichi, Japan; hisanori.muto@fujita-hu.ac.jp (H.M.); ytachi@fujita-hu.ac.jp (Y.T.); gakushi.komura@fujita-hu.ac.jp (G.K.); tkjnkn@fujita-hu.ac.jp (T.N.); hiroyuki.tanaka@fujita-hu.ac.jp (H.T.); knakaoka@fujita-hu.ac.jp (K.N.); k-funa@fujita-hu.ac.jp (K.F.); nmitsu@fujita-hu.ac.jp (M.N.); ryoji.miyahara@fujita-hu.ac.jp (R.M.); eizaburo.ono@fujita-hu.ac.jp (E.O.); 2 Department of Gastroenterology and Hepatology, Fujita Health University Bantane Hospital, Nagoya 454-8509, Aichi, Japan; 3 Department of Gastroenterology, Fujita Health University Okazaki Medical Center, Okazaki 444-0827, Aichi, Japan

**Keywords:** hepatocellular carcinoma, lenvatinib, immune checkpoint inhibitor, atezolizumab plus bevacizumab, durvalumab plus tremelimumab, sequential therapy, VEGF inhibition, treatment resistance

## Abstract

**Background/Objectives:** The efficacy of lenvatinib after different first-line immune checkpoint inhibitor (ICI)-based regimens for hepatocellular carcinoma (HCC) remains unclear. **Materials and Methods:** In this retrospective single-center study, we analyzed 56 patients with unresectable HCC who received lenvatinib as second-line therapy after atezolizumab plus bevacizumab (Atz/Bev; *n* = 41) or durvalumab plus tremelimumab (Dur/Tre; *n* = 15). Antitumor response was evaluated using RECIST v1.1 and modified RECIST (mRECIST). **Results:** Patients in the Dur/Tre group demonstrated significantly lower disease control rates (46.7% vs. 82.9%, *p* = 0.014) and shorter progression-free survival (PFS) (median, 56 vs. 210 days; *p* = 0.015) during prior ICI therapy, indicating more refractory disease. Despite this, lenvatinib demonstrated clinically meaningful antitumor activity after both Atz/Bev and Dur/Tre. Objective response rates were 12.2% vs. 26.7% by RECIST v1.1 and 41.5% vs. 60.0% by mRECIST in the Atz/Bev and Dur/Tre groups, respectively. Similar findings were observed in the Child–Pugh class A subgroup, in which mRECIST response rates were numerically higher in the Dur/Tre group. PFS and overall survival (OS) after lenvatinib initiation were comparable between groups. Objective responses were observed even in patients with progressive disease during prior ICI therapy. Multivariate analysis identified ECOG performance status and mALBI grade, but not prior ICI regimen or antitumor response, as independent prognostic factors for OS. **Conclusions:** Lenvatinib demonstrated clinically meaningful efficacy after both Atz/Bev and Dur/Tre, and no significant association was observed between prior ICI response and subsequent lenvatinib efficacy. These findings suggest that resistance to immunotherapy does not necessarily confer resistance to lenvatinib in patients with unresectable HCC. The observed differences in response patterns according to prior treatment exposure warrant further investigation and may have implications for treatment sequencing in the current ICI era. However, these findings should be considered hypothesis-generating and require validation in larger multicenter studies.

## Simple Summary

Sequential treatment after immune checkpoint inhibitor therapy remains a major challenge in unresectable hepatocellular carcinoma (HCC). Whether the efficacy of lenvatinib differs according to prior first-line immune checkpoint inhibitor regimens remains unclear. We evaluated the efficacy of second-line lenvatinib after atezolizumab plus bevacizumab or durvalumab plus tremelimumab in real-world clinical practice. Lenvatinib retained clinically meaningful antitumor activity after prior immunotherapy, including in patients with progressive disease during first-line treatment. Interestingly, numerically greater tumor shrinkage was observed in patients without prior exposure to VEGF inhibition during lenvatinib treatment. These findings suggest that prior resistance to immunotherapy does not necessarily preclude subsequent antitumor activity of lenvatinib and highlight the importance of treatment sequencing and preservation of liver function in HCC management.

## 1. Introduction

Recent advances in immune checkpoint inhibitor (ICI)-based combination therapies have dramatically changed the treatment landscape of unresectable hepatocellular carcinoma (HCC) [1]. Atezolizumab plus bevacizumab (Atz/Bev) and durvalumab plus tremelimumab (Dur/Tre) are now widely used as first-line treatment options and have demonstrated significant survival benefits compared with sorafenib [2,3]. These advances reflect the growing role of immunotherapy as a cornerstone of modern cancer treatment [4].

Despite these advances, most patients eventually experience progressive disease (PD) and require subsequent systemic therapy. Therefore, optimization of sequential treatment strategies has become a critical clinical challenge in the current ICI era. However, robust evidence regarding optimal second-line treatment after ICI-based therapy remains limited. Current treatment algorithms recommend several molecular targeted agents in parallel after failure of first-line immunotherapy, largely irrespective of the specific prior ICI regimen, because prospective comparative data regarding the optimal therapeutic sequence are lacking [1,5,6,7,8]. In real-world clinical practice in Japan, lenvatinib has emerged as one of the most frequently used second-line agents after ICI-based therapy because of its high antitumor activity and manageable safety profile [9].

Lenvatinib [10], a multikinase inhibitor targeting vascular endothelial growth factor receptor (VEGFR)1–3 and fibroblast growth factor receptor (FGFR)1–4 signaling pathways, is commonly used after ICI-based therapy and has demonstrated favorable antitumor activity in real-world clinical practice [11,12,13,14,15]. However, clinical data regarding its efficacy after different first-line ICI regimens remain limited. In particular, evidence regarding lenvatinib after Dur/Tre is extremely scarce [16], with only limited real-world data available to date.

Importantly, Atz/Bev and Dur/Tre differ substantially in their therapeutic and biologic backgrounds, especially with regard to prior exposure to VEGF inhibition. These differences may potentially influence the tumor immune microenvironment and subsequent sensitivity to molecular targeted therapies [17,18]. Therefore, whether lenvatinib demonstrates comparable efficacy after these distinct first-line regimens remains an important unresolved clinical question. Furthermore, it remains unclear whether resistance to prior ICI therapy influences the subsequent efficacy of lenvatinib. Clarifying these issues is clinically relevant because many patients experience PD during first-line immunotherapy and still require effective sequential treatment options. Although several previous studies have reported the efficacy of lenvatinib after failure of ICI-based therapy, most have focused on patients previously treated with Atz/Bev. Consequently, the efficacy of lenvatinib after Dur/Tre, as well as the potential influence of prior ICI response and prior VEGF inhibitor exposure on subsequent lenvatinib efficacy, remains insufficiently understood. We therefore hypothesized that prior ICI response and differences in prior VEGF inhibitor exposure might influence subsequent response patterns to lenvatinib.

The aim of this study was to evaluate the efficacy of lenvatinib as second-line therapy after ICI-based treatment in patients with unresectable HCC, with a particular focus on differences according to prior first-line regimen, including Atz/Bev and Dur/Tre. In addition, we investigated the relationship between response to prior ICI therapy and subsequent antitumor response to lenvatinib.

## 2. Materials and Methods

### 2.1. Study Design and Patients

This retrospective cohort study included consecutive patients with unresectable HCC who received lenvatinib as second-line therapy following discontinuation of first-line ICI-based therapy at our institution. In some patients who discontinued first-line ICI-based therapy because of adverse events (AEs), lenvatinib was initiated after radiological progression had been confirmed during follow-up. The study protocol was approved by the Institutional Review Board of Fujita Health University (HM24-418; 16 December 2024) and conducted in accordance with the Declaration of Helsinki. Between October 2020 and February 2026, a total of 111 patients received Atz/Bev and 42 received Dur/Tre as first-line systemic therapy. Among these patients, 41 and 15, respectively, subsequently received lenvatinib as second-line therapy and were included in the present analysis. Patients were stratified according to prior first-line treatment (Atz/Bev vs. Dur/Tre), which differed in exposure to VEGF inhibition during first-line therapy.

### 2.2. Treatment Protocol

Lenvatinib was generally administered according to body weight (12 mg/day for patients weighing ≥60 kg and 8 mg/day for those weighing <60 kg) [10]. Dose modifications were permitted at the discretion of the treating physician based on tolerability, hepatic reserve, and general condition. In real-world clinical practice, some patients started lenvatinib at a reduced dose level because of prior treatment-related AEs, impaired hepatic reserve, poor performance status, or concerns regarding overall tolerability. Treatment was continued until radiological PD, unacceptable toxicity, or clinical deterioration at the discretion of the treating physician.

### 2.3. Evaluation and Definitions

Antitumor response was evaluated using contrast-enhanced computed tomography or magnetic resonance imaging and assessed according to Response Evaluation Criteria in Solid Tumors version 1.1 (RECIST v1.1) and modified Response Evaluation Criteria in Solid Tumors (mRECIST) [19,20]. The first radiological assessment was generally performed 6 weeks after initiation of lenvatinib, followed by repeated imaging evaluations every 4–12 weeks depending on the clinical condition of each patient. AEs were graded according to the Common Terminology Criteria for Adverse Events (CTCAE), version 5.0 [21]. Both RECIST v1.1 and mRECIST were used because these criteria provide complementary information regarding treatment response in HCC. RECIST v1.1 is the standard method for evaluating changes in tumor size and has been widely used in clinical studies of ICIs. In contrast, mRECIST assesses viable enhancing tumor tissue and may better reflect the antiangiogenic effects of lenvatinib, which can induce tumor devascularization without substantial changes in overall tumor size.

Progression-free survival (PFS) was defined as the time from initiation of lenvatinib to radiological progression or death from any cause, and overall survival (OS) was defined as the time from initiation of lenvatinib to death from any cause. Objective response rate (ORR) and disease control rate (DCR) were defined as the proportions of patients achieving complete response (CR) or partial response (PR), and CR, PR, or stable disease (SD), respectively. The PFS of prior ICI therapy was defined as the time from initiation of first-line ICI treatment to PD. Time to treatment discontinuation was defined as the time from initiation of lenvatinib to treatment discontinuation for any reason. In addition, transition analyses of tumor response from prior ICI therapy to second-line lenvatinib were performed using both RECIST v1.1 and mRECIST to evaluate the relationship between prior ICI response and subsequent lenvatinib efficacy.

The primary analyses were performed in the overall cohort, which included all patients who received second-line lenvatinib after first-line ICI-based therapy. Because hepatic reserve strongly influences treatment outcomes in HCC, additional analyses were also performed in the Child–Pugh class A subgroup at lenvatinib initiation. In this subgroup, antitumor response, PFS, OS, and time to treatment discontinuation were evaluated according to prior first-line ICI regimen.

The relative dose intensity (RDI) of lenvatinib was evaluated during the first 6 weeks after treatment initiation. RDI was calculated as the cumulative dose actually administered divided by the planned cumulative dose according to the recommended dose based on body weight (12 mg/day for patients weighing ≥60 kg and 8 mg/day for patients weighing <60 kg). In patients who died within 6 weeks, RDI was calculated using the actual treatment period.

### 2.4. Statistical Analysis

Continuous variables were compared using the Mann–Whitney U test, and categorical variables were compared using Fisher’s exact test. Survival curves were estimated using the Kaplan–Meier method and compared using the log-rank test. All statistical analyses were performed using EZR (Saitama Medical Center, Jichi Medical University, Saitama, Japan), a graphical user interface for R [22]. A *p*-value < 0.05 was considered statistically significant. Variables with *p* values < 0.10 in univariate analysis and clinically relevant factors were entered into the multivariate Cox proportional hazards model to estimate hazard ratios (HRs) and 95% confidence intervals (CIs). To avoid multicollinearity and overfitting, variables reflecting similar clinical domains were not included simultaneously in the multivariate analysis. The same comparative analyses were repeated in the Child–Pugh class A subgroup to reduce the potential confounding effect of impaired hepatic reserve.

## 3. Results

### 3.1. Baseline Characteristics

**Figure 1:**
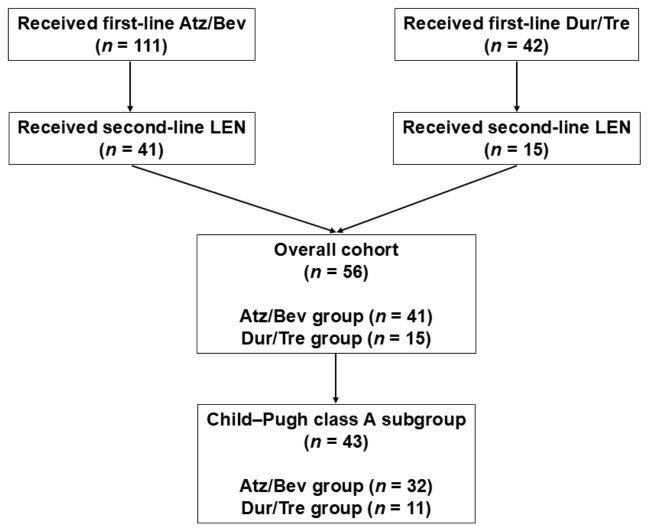
Patient flow diagram. The diagram illustrates patient selection and the relationship between the overall cohort (*n* = 56) and the Child–Pugh class A subgroup (*n* = 43).

As shown in Figure 1, a total of 56 patients who received second-line lenvatinib after first-line Atz/Bev (*n* = 41) or Dur/Tre (*n* = 15) were included in the overall cohort. Among them, 43 patients with Child–Pugh class A liver function at lenvatinib initiation (Atz/Bev, *n* = 32; Dur/Tre, *n* = 11) comprised the Child–Pugh class A subgroup.

Baseline characteristics at the initiation of second-line lenvatinib in the overall cohort are summarized in Table 1. Patients were stratified according to prior first-line treatment (Atz/Bev vs. Dur/Tre). In the overall cohort, baseline characteristics including liver function and tumor burden were generally comparable between the two groups, with no significant differences in patient demographics, hepatic reserve, tumor characteristics, or tumor marker levels. In contrast, significant differences were observed in the clinical course during prior ICI therapy. The disease control rate with prior ICI therapy was significantly lower in the Dur/Tre group than in the Atz/Bev group (46.7% vs. 82.9%, *p* = 0.014). In addition, PFS during prior ICI therapy was significantly shorter in the Dur/Tre group (median, 56 vs. 210 days; *p* = 0.015). The median follow-up time after lenvatinib initiation was 7.5 months (range, 0.4–58.9 months) in the overall cohort, 7.9 months (range, 0.4–58.9 months) in the Atz/Bev group, and 6.7 months (range, 0.5–16.7 months) in the Dur/Tre group.

Baseline characteristics at the initiation of second-line lenvatinib in the Child–Pugh class A subgroup are summarized in Table 2. Similar baseline characteristics were observed in this subgroup. The Dur/Tre group showed numerically lower disease control rates and shorter prior ICI PFS than the Atz/Bev group, although these differences did not reach statistical significance.

### 3.2. Antitumor Response to Lenvatinib

Antitumor responses to second-line lenvatinib in the overall cohort are summarized in Table 3. According to RECIST v1.1, the ORR and DCR in the overall cohort were 16.1% and 78.6%, respectively. There were no significant differences between the Atz/Bev and Dur/Tre groups in ORR (12.2% vs. 26.7%, *p* = 0.229) or DCR (80.5% vs. 73.3%, *p* = 0.715). Based on mRECIST, the ORR and DCR in the overall cohort were 46.4% and 78.6%, respectively. The ORR remained comparable between the Atz/Bev and Dur/Tre groups (41.5% vs. 60.0%, *p* = 0.243), while the DCR was unchanged from that observed using RECIST v1.1.

Antitumor responses in the Child–Pugh class A subgroup at lenvatinib initiation are summarized in Table 4. According to RECIST, the ORR was higher in the Dur/Tre group than in the Atz/Bev group (36.4% vs. 15.6%, *p* = 0.201), whereas the DCR was similar between the two groups (81.3% vs. 81.8%, *p* = 1.000). Based on mRECIST, the ORR in the Dur/Tre group was higher than that in the Atz/Bev group (72.7% vs. 37.5%, *p* = 0.078), whereas the DCR was similar between the two groups (81.3% vs. 81.8%, *p* = 1.000).

### 3.3. Survival Outcomes

**Figure 2:**
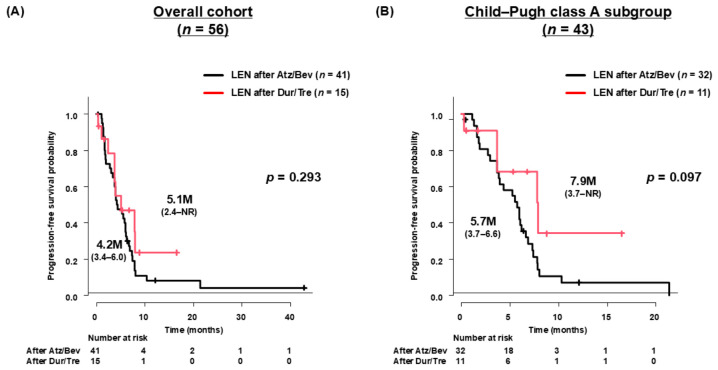
Kaplan–Meier curves for progression-free survival (PFS) according to prior immune checkpoint inhibitor (ICI) regimen. (**A**) Overall cohort; (**B**) Child–Pugh class A subgroup. Patients were stratified according to prior treatment with atezolizumab plus bevacizumab (Atz/Bev) or durvalumab plus tremelimumab (Dur/Tre). *p* values were calculated using the log-rank test.

PFS after initiation of lenvatinib according to prior ICI regimen is shown in Figure 2. In the overall cohort, there were no significant differences in PFS between the Atz/Bev and Dur/Tre groups (*p* = 0.2928). Similarly, in the Child–Pugh class A subgroup, PFS did not significantly differ between the two groups, although median PFS was numerically longer in the Dur/Tre group (*p* = 0.0974).

**Figure 3:**
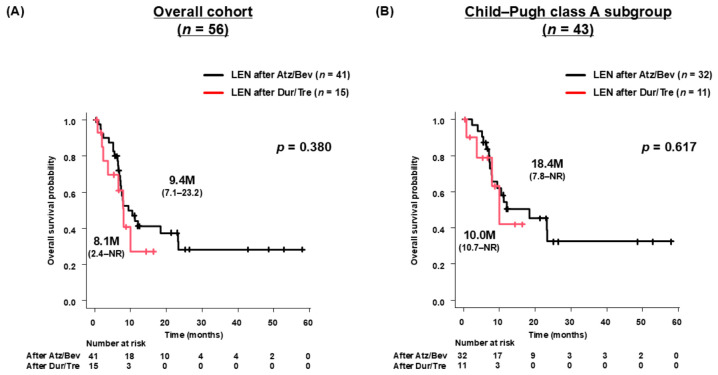
Kaplan–Meier curves for overall survival (OS) according to prior immune checkpoint inhibitor (ICI) regimen. (**A**) Overall cohort; (**B**) Child–Pugh class A subgroup. Patients were stratified according to prior treatment with Atz/Bev or Dur/Tre. *p* values were calculated using the log-rank test.

OS after initiation of lenvatinib according to prior ICI regimen is shown in Figure 3. OS also did not significantly differ between the Atz/Bev and Dur/Tre groups in either the overall cohort (*p* = 0.3802) or the Child–Pugh class A subgroup (*p* = 0.6167).

### 3.4. Transition of Tumor Response

**Figure 4:**
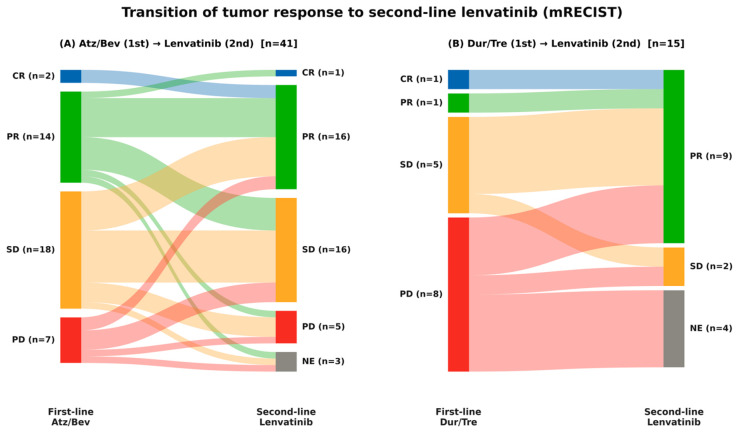
Transition of tumor response from prior immune checkpoint inhibitor therapy to second-line lenvatinib according to mRECIST in the overall cohort. (**A**) Patients previously treated with Atz/Bev. (**B**) Patients previously treated with Dur/Tre. The left side indicates best response to first-line ICI therapy, and the right side indicates best response to second-line lenvatinib. The width of each flow is proportional to the number of patients transitioning between response categories. Numbers within the flows indicate the number of patients in each transition pathway.

**Figure 5:**
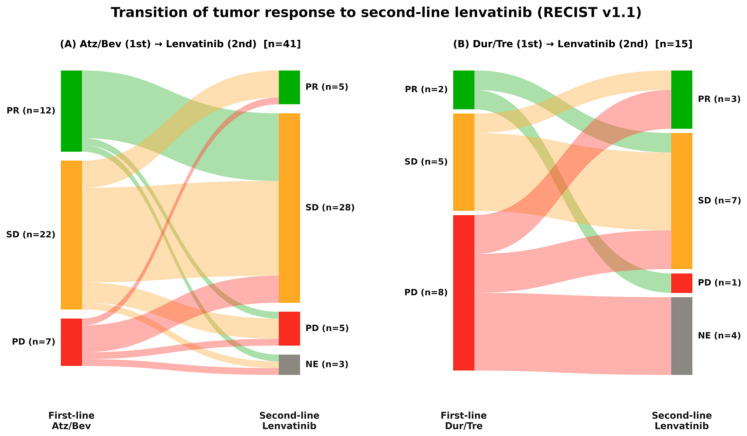
Transition of tumor response from prior immune checkpoint inhibitor therapy to second-line lenvatinib according to RECIST v1.1 in the overall cohort. (**A**) Patients previously treated with Atz/Bev. (**B**) Patients previously treated with Dur/Tre. The left side indicates best response to first-line ICI therapy, and the right side indicates best response to second-line lenvatinib. The width of each flow is proportional to the number of patients transitioning between response categories. Numbers within the flows indicate the number of patients in each transition pathway.

Transition analyses of tumor response according to prior ICI response category in the overall cohort are shown in Figure 4 and Figure 5. Figure 4 presents mRECIST-based analyses, whereas Figure 5 presents RECIST v1.1-based analyses. In both figures, Sankey diagrams were used to illustrate transitions between response categories during prior ICI therapy and subsequent lenvatinib treatment.

Objective responses and disease control with lenvatinib were observed even among patients with PD during prior ICI therapy, suggesting that prior resistance to ICI therapy may not necessarily preclude subsequent lenvatinib efficacy. In the Atz/Bev group, disease control with lenvatinib was achieved in 4 of 7 patients with prior PD during ICI therapy, including one patient who achieved PR according to mRECIST. Similarly, in the Dur/Tre group, objective responses including PR were also observed despite prior treatment failure. Overall, broadly similar transition patterns were observed using both RECIST v1.1 and mRECIST criteria.

### 3.5. Prognostic Analysis

Univariate and multivariate analyses for PFS after initiation of lenvatinib in the overall cohort are summarized in Table 5. No independent prognostic factors for PFS were identified in multivariate analysis. Importantly, neither prior first-line ICI regimen nor prior antitumor response to ICI therapy was significantly associated with PFS after lenvatinib initiation.

Univariate and multivariate analyses for OS after initiation of lenvatinib in the overall cohort are summarized in Table 6. Multivariate analysis for OS identified ECOG performance status and mALBI grade as independent prognostic factors. ECOG PS 0 was independently associated with favorable OS (HR, 0.294; 95% CI, 0.123–0.701; *p* = 0.006), and mALBI grade 1/2a was also significantly associated with favorable OS (HR, 0.169; 95% CI, 0.075–0.381; *p* < 0.001). In contrast, prior first-line ICI regimen and prior antitumor response to ICI therapy were not independently associated with OS.

### 3.6. Safety

**Figure 6:**
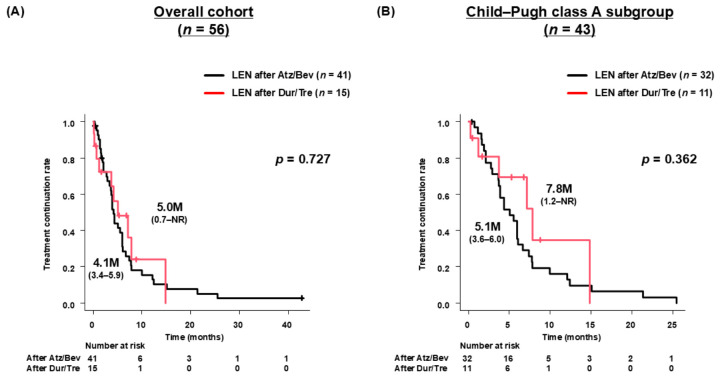
Kaplan–Meier curves for time to treatment discontinuation according to prior ICI regimen. (**A**) Overall cohort; (**B**) Child–Pugh class A subgroup. *p* values were calculated using the log-rank test.

Time to treatment discontinuation of lenvatinib according to prior ICI regimen is shown in Figure 6. No significant differences were observed between the Atz/Bev and Dur/Tre groups in either the overall cohort (*p* = 0.7268) or the Child–Pugh class A subgroup (*p* = 0.3617).

The overall safety profile of lenvatinib was consistent with its known toxicity profile, and no unexpected AEs were observed (Table 7). The most common AEs were appetite loss (50.0%), general fatigue (46.4%), and proteinuria (33.9%). Grade ≥ 3 AEs were relatively infrequent, with proteinuria being the most common severe AE (12.5%). Overall, the incidence and severity of AEs were generally comparable between the two groups. However, proteinuria was significantly more frequently observed in patients previously treated with Atz/Bev than in those previously treated with Dur/Tre (43.9% vs. 6.7%, *p* = 0.0105).

The 6-week RDI of lenvatinib was comparable between the Atz/Bev and Dur/Tre groups in both the overall cohort (50.0% vs. 40.0%, *p* = 0.170) and the Child–Pugh class A subgroup (53.0% vs. 46.0%, *p* = 0.362) (Table 8). No significant differences in treatment exposure were observed between the two groups.

Post-lenvatinib treatment according to prior ICI regimen is summarized in Table 9. In the overall cohort, subsequent treatment after lenvatinib discontinuation was administered to 24 of 32 patients (75.0%) in the Atz/Bev group and 3 of 7 patients (42.9%) in the Dur/Tre group. In the Child–Pugh class A subgroup, the corresponding proportions were 77.8% and 66.7%, respectively.

## 4. Discussion

In this study, we evaluated the efficacy of lenvatinib as second-line therapy after different first-line ICI-based therapies in patients with unresectable HCC. The major findings were as follows: (1) patients previously treated with Dur/Tre exhibited more refractory disease during prior ICI therapy, as reflected by lower DCRs and shorter PFS; (2) despite these differences, no significant differences in the antitumor efficacy or survival outcomes of subsequent lenvatinib were observed according to prior ICI regimen; and (3) ORRs to lenvatinib were observed even in patients with PD during prior ICI therapy, suggesting that prior resistance to immunotherapy may not necessarily preclude subsequent antitumor activity of lenvatinib.

The preserved efficacy of lenvatinib after ICI failure may be explained by its distinct mechanism of action. Unlike immunotherapy, lenvatinib exerts antitumor effects primarily through inhibition of angiogenic and proliferative signaling pathways, including VEGFR and FGFR [17,18]. Preclinical studies have demonstrated that lenvatinib inhibits multiple signaling pathways beyond VEGF signaling, particularly FGFR-mediated pathways, which may remain active despite resistance to immune checkpoint inhibition [23,24]. Therefore, tumor resistance to ICI therapy may not directly confer resistance to lenvatinib [11,12,13]. Taken together with previous preclinical and translational studies, our findings are consistent with the concept of a biologic dissociation between resistance to immunotherapy and sensitivity to molecular targeted therapy in HCC. Clinically, this observation is important because many patients experience PD during first-line ICI therapy but still require effective sequential treatment options [5,6].

Interestingly, although the Dur/Tre group demonstrated poorer disease control during prior ICI therapy, subsequent lenvatinib treatment resulted in higher response rates, particularly according to mRECIST evaluation in patients with Child–Pugh class A liver function. Because mRECIST primarily reflects changes in tumor vascularity and viable tumor components [20], these findings may suggest that prior exposure to anti-VEGF therapy influences subsequent response patterns to lenvatinib [17,18]. In contrast to patients previously treated with Atz/Bev, those treated with Dur/Tre were VEGF inhibitor-naïve before lenvatinib initiation, which may have contributed to the greater degree of tumor shrinkage observed in this subgroup. One possible explanation for the numerically greater tumor shrinkage observed in the Dur/Tre group may be the absence of prior VEGF inhibitor exposure. Because all patients in the Atz/Bev group had previously received bevacizumab whereas those in the Dur/Tre group were VEGF inhibitor-naïve, differences in response patterns may reflect prior anti-VEGF exposure rather than differences in the immunotherapy regimen itself. However, no significant differences were observed in the 6-week relative dose intensity or time to treatment discontinuation between groups, suggesting that treatment exposure alone is unlikely to fully explain the observed response patterns. Therefore, the observed differences in response patterns should be interpreted cautiously and regarded as hypothesis-generating.

Transition analyses further supported these findings. Objective responses and disease control with lenvatinib were observed even among patients with prior PD during ICI therapy. These findings suggest that poor response to first-line immunotherapy should not necessarily preclude consideration of subsequent lenvatinib treatment [11,12,13,16]. Rather, lenvatinib may retain clinically meaningful antitumor activity even in patients who exhibit primary resistance to ICI therapy.

Another important finding was that no independent association was observed between prior ICI regimen or prior antitumor response and either PFS or OS after lenvatinib initiation. In contrast, ECOG performance status and mALBI grade remained significant prognostic factors for OS. These findings suggest that host-related factors, particularly hepatic reserve and general condition, may have a greater impact on long-term outcomes after sequential therapy than prior sensitivity to immunotherapy itself [25,26,27,28]. In the current ICI era, maintaining hepatic reserve and preserving eligibility for subsequent therapy may be more important than maximizing the efficacy of a single treatment line.

Overall, the efficacy outcomes observed in the present study were generally consistent with those reported in previous studies evaluating lenvatinib after ICI-based therapy. Collectively, these findings further support the potential role of lenvatinib as a clinically relevant treatment option following ICI-based first-line therapy in patients with unresectable HCC, although further validation in larger prospective studies is warranted.

Although our findings are broadly consistent with previous reports, the present study differs from our previous study [12] evaluating lenvatinib after early progression during Atz/Bev therapy in several important respects. First, the current study included patients treated with both Atz/Bev and Dur/Tre, allowing comparison according to prior ICI regimen and prior VEGF inhibitor exposure. Second, we evaluated the relationship between response to prior ICI therapy and subsequent lenvatinib efficacy using transition analyses based on both RECIST v1.1 and mRECIST. Third, the present study provides novel insights into treatment sequencing in the current era in which multiple first-line ICI-based regimens are available.

This study has several limitations. First, this was a retrospective single-center study with a relatively small sample size, particularly in the Dur/Tre group. Therefore, selection bias cannot be excluded, the generalizability of the findings may be limited, and the statistical power to detect clinically meaningful differences between treatment groups may have been insufficient. Second, treatment strategies, including dose modifications, treatment timing, and subsequent therapies, were not standardized. In some patients who discontinued first-line ICI-based therapy because of adverse events, lenvatinib was initiated after radiological progression had been confirmed during follow-up as part of routine clinical practice. Therefore, the interval before lenvatinib initiation may have been longer in such cases, potentially affecting patient condition at treatment initiation. In addition, post-lenvatinib treatment strategies were heterogeneous, and differences in subsequent therapy may have influenced overall survival outcomes. Third, because nivolumab plus ipilimumab became available relatively recently during the study period in Japan, this regimen could not be adequately evaluated. Finally, the relatively small number of patients in the Dur/Tre group may have limited the statistical power to detect clinically meaningful differences between treatment groups. Therefore, the absence of statistically significant differences should not be interpreted as evidence of equivalent efficacy. Further multicenter prospective studies are warranted to validate our findings.

Despite these limitations, our study provides clinically meaningful insights into sequential treatment strategies for HCC in the current ICI era. The observed differences in response patterns according to prior treatment exposure warrant further investigation and may have implications for treatment sequencing in HCC. Future multicenter prospective studies are needed to validate these findings and to clarify whether prior ICI response or prior VEGF inhibitor exposure can be used to optimize treatment selection and sequencing after first-line immunotherapy.

## 5. Conclusions

Lenvatinib demonstrated clinically meaningful antitumor activity as second-line therapy after both Atz/Bev and Dur/Tre, and no significant association was observed between prior treatment response and subsequent lenvatinib efficacy. These findings suggest that resistance to immunotherapy does not necessarily confer resistance to lenvatinib in patients with unresectable HCC. The observed differences in response patterns according to prior treatment exposure may have implications for treatment sequencing in the current ICI era; however, given the retrospective design and relatively small sample size, particularly in the Dur/Tre group, these findings should be considered hypothesis-generating and require validation in larger multicenter studies.

## Figures and Tables

**Table 1:** Baseline characteristics at initiation of second-line lenvatinib according to prior ICI regimen in the overall cohort.

Characteristics	Overall Cohort (*n* = 56)	After Atz/Bev (*n* = 41)	After Dur/Tre (*n* = 15)	*p*-Value
Age, years, median (range)	76 (40–90)	74 (42–90)	76 (40–88)	0.941
Sex, male/female	44/12	31/10	13/2	0.481
Etiology, HBV/HCV/non-viral	6/8/42	3/7/31	3/1/11	
Etiology, non-viral, *n*, %	42 (75.0)	31 (75.6)	11 (73.3)	
ECOG PS, 0/1/2	42/12/2	29/11/1	13/1/1	
ECOG PS 0, *n* (%)	42 (75.0)	29 (70.7)	13 (86.7)	0.308
Child–Pugh score, 5/6/7/8/9	30/13/7/2/4	23/9/5/2/2	7/4/2/0/2	
Child–Pugh class, A/B	43/13	32/9	11/4	0.730
ALBI score, median (range)	−2.31 (−3.35 to −1.01)	−2.31 (−3.10 to −1.05)	−2.02 (−3.35 to −1.01)	0.598
mALBI grade, 1/2a/2b/3	12/19/20/5	10/14/14/3	2/5/6/2	
BCLC stage, B/C	26/30	18/23	8/7	0.561
Number of tumors, <4/≥4	12/44	7/34	5/10	0.270
Tumor size, <50/≥50 mm	33/23	24/17	9/6	1.000
Portal vein invasion, absent/present	39/17	28/13	11/4	1.000
Extrahepatic spread, absent/present	33/23	25/16	8/7	0.761
AFP, median (range), ng/mL	115.8 (1.3–5,726,000)	112.0 (2.7–5,726,000)	192.0 (1.3–55,835)	0.579
AFP, <400/≥400 ng/mL	37/19	28/13	9/6	0.751
DCP, median (range), mAU/mL	2576 (18–235,659)	2598 (18–235,659)	2226 (142–42,956)	0.904
DCP, <400/≥400 mAU/mL	13/43	9/32	4/11	0.730
UPCR, median (range)	178 (0–4168)	256 (0–4168)	115 (33–1238)	0.313
UPCR, <2000/≥2000	50/6	35/6	15/0	0.177
Prior ICI best response (RECIST v1.1), CR/PR/SD/PD	0/14/27/15	0/12/22/7	0/2/5/8	
Disease control with prior ICI, *n* (%)	41 (73.2)	34 (82.9)	7 (46.7)	0.014
Reason for discontinuation of prior ICI, PD/AE	45/11	31/10	14/1	0.255
PFS of prior ICI, days, median (range)	196 (27–805)	210 (92–805)	56 (27–356)	0.015
Initial lenvatinib dose, 12/8/4 mg	20/22/14	16/16/10	6/5/4	
Reduced starting dose, yes/no	26/30	20/21	6/9	0.763

Abbreviations: ICI, immune checkpoint inhibitor; Atz/Bev, atezolizumab plus bevacizumab; Dur/Tre, durvalumab plus tremelimumab; HBV, hepatitis B virus; HCV, hepatitis C virus; ECOG PS, Eastern Cooperative Oncology Group performance status; ALBI, albumin–bilirubin; mALBI, modified albumin–bilirubin; BCLC, Barcelona Clinic Liver Cancer; AFP, alpha-fetoprotein; DCP, des-gamma-carboxy prothrombin; UPCR, urine protein-to-creatinine ratio; RECIST, Response Evaluation Criteria in Solid Tumors; CR, complete response; PR, partial response; SD, stable disease; PD, progressive disease; AE, adverse event; PFS, progression-free survival.

**Table 2:** Baseline characteristics at initiation of second-line lenvatinib according to prior ICI regimen in the Child–Pugh class A subgroup.

Characteristics	Child–Pugh Class A Subgroup (*n* = 43)	After Atz/Bev (*n* = 32)	After Dur/Tre (*n* = 11)	*p*-Value
Age, years, median (range)	77 (42–90)	76 (42–90)	77 (65–88)	0.512
Sex, male/female	33/10	24/8	9/2	1.000
Etiology, HBV/HCV/non-viral	4/8/31	2/7/23	2/2/8	
Etiology, non-viral, *n*, %	31 (72.1)	23 (71.9)	8 (72.7)	0.727
ECOG PS, 0/1/2	35/7/1	25/7/0	10/0/1	
ECOG PS 0, *n* (%)	35 (81.4)	25 (78.1)	10 (90.9)	0.656
Child–Pugh score, 5/6	30/13	23/9	7/4	0.709
ALBI score, median (range)	−2.38 (−3.35 to −1.62)	−2.36 (−3.10 to −1.62)	−2.24 (−3.35 to −1.88)	0.889
mALBI grade, 1/2a/2b	12/19/12	10/14/8	2/5/4	
BCLC stage, B/C	19/24	13/19	6/5	0.495
Number of tumors, <4/≥4	11/32	7/25	4/7	0.430
Tumor size, <50/≥50 mm	26/17	19/13	7/4	1.000
Portal vein invasion, absent/present	30/13	22/10	8/3	1.000
Extrahepatic spread, absent/present	25/18	19/13	6/5	1.000
AFP, median (range), ng/mL	98.0 (1.7–5,726,000)	74.6 (2.7–5,726,000)	192.0 (1.7–30,009)	0.428
AFP, <400/≥400 ng/mL	30/13	23/9	7/4	0.709
DCP, median (range), mAU/mL	2598 (18–43,812)	2248 (18–43,812)	3597 (142–28,597)	0.626
DCP, <400/≥400 mAU/mL	11/32	8/24	3/8	1.000
UPCR, median (range)	174 (0–3260)	210 (0–3260)	174 (33–1238)	0.504
UPCR, <2000/≥2000	39/4	28/4	11/0	0.558
Prior ICI best response (RECIST v1.1), CR/PR/SD/PD	0/13/17/13	0/7/14/7	0/2/3/6	
Disease control with prior ICI, n (%)	30 (69.8)	21 (65.6)	5 (45.5)	0.061
Reason for discontinuation of prior ICI, PD/AE	34/9	24/8	10/1	0.407
PFS of prior ICI, days, median (range)	203 (27–805)	209 (42–805)	56 (27–356)	0.056
Initial lenvatinib dose, 12/8/4 mg	16/18/9	12/13/7	4/5/2	
Reduced starting dose, yes/no	18/25	14/18	4/7	0.739

Abbreviations: ICI, immune checkpoint inhibitor; Atz/Bev, atezolizumab plus bevacizumab; Dur/Tre, durvalumab plus tremelimumab; HBV, hepatitis B virus; HCV, hepatitis C virus; ECOG PS, Eastern Cooperative Oncology Group performance status; ALBI, albumin–bilirubin; mALBI, modified albumin–bilirubin; BCLC, Barcelona Clinic Liver Cancer; AFP, alpha-fetoprotein; DCP, des-gamma-carboxy prothrombin; UPCR, urine protein-to-creatinine ratio; RECIST, Response Evaluation Criteria in Solid Tumors; CR, complete response; PR, partial response; SD, stable disease; PD, progressive disease; AE, adverse event; PFS, progression-free survival.

**Table 3:** Antitumor response to second-line lenvatinib according to prior ICI regimen (RECIST v1.1 and mRECIST) in the overall cohort.

	Overall Cohort (*n* = 56)	After Atz/Bev (*n* = 41)	After Dur/Tre (*n* = 15)	*p*-Value
RECIST v1.1, *n* (CR/PR/SD/PD/NE)	0/9/35/5/7	0/5/28/5/3	0/4/7/0/4	
ORR, *n* (%)	9 (16.1)	5 (12.2)	4 (26.7)	0.229
DCR, *n* (%)	44 (78.6)	33 (80.5)	11 (73.3)	0.715
mRECIST, *n* (CR/PR/SD/PD/NE)	1/25/18/5/7	1/16/16/5/3	0/9/2/0/4	
ORR, *n* (%)	26 (46.4)	17 (41.5)	9 (60.0)	0.243
DCR, *n* (%)	44 (78.6)	33 (80.5)	11 (73.3)	0.715

Abbreviations: ICI, immune checkpoint inhibitor; Atz/Bev, atezolizumab plus bevacizumab; Dur/Tre, durvalumab plus tremelimumab; RECIST v1.1, Response Evaluation Criteria in Solid Tumors version 1.1; mRECIST, modified RECIST; ORR, objective response rate; DCR, disease control rate; CR, complete response; PR, partial response; SD, stable disease; PD, progressive disease; NE, not evaluable.

**Table 4:** Antitumor response to second-line lenvatinib according to prior ICI regimen (RECIST v1.1 and mRECIST) in the Child–Pugh class A subgroup.

	Child–Pugh Class A Subgroup (*n* = 43)	After Atz/Bev (*n* = 32)	After Dur/Tre (*n* = 11)	*p*-Value
RECIST v1.1, *n* (CR/PR/SD/PD/NE)	0/9/26/4/4	0/5/21/4/2	0/4/5/0/2	
ORR, *n* (%)	9 (20.9)	5 (15.6)	4 (36.4)	0.201
DCR, *n* (%)	35 (81.4)	26 (81.3)	9 (81.8)	1.000
mRECIST, *n* (CR/PR/SD/PD/NE)	1/19/15/4/4	1/11/14/4/2	0/8/1/0/2	
ORR, *n* (%)	20 (46.5)	12 (37.5)	8 (72.7)	0.078
DCR, *n* (%)	35 (81.4)	26 (81.3)	9 (81.8)	1.000

Abbreviations: ICI, immune checkpoint inhibitor; Atz/Bev, atezolizumab plus bevacizumab; Dur/Tre, durvalumab plus tremelimumab; RECIST v1.1, Response Evaluation Criteria in Solid Tumors version 1.1; mRECIST, modified RECIST; ORR, objective response rate; DCR, disease control rate; CR, complete response; PR, partial response; SD, stable disease; PD, progressive disease; NE, not evaluable.

**Table 5:** Univariate and multivariate analyses of prognostic factors for PFS after initiation of second-line lenvatinib in the overall cohort.

	Univariate Analysis		Multivariate Analysis	
Factors	HR (95% CI)	*p* Value	HR (95% CI)	*p* Value
Age (≥75 years)	0.571 (0.317–1.026)	0.061	0.683 (0.360–1.298)	0.245
Sex (female)	1.005 (0.496–2.034)	0.990		
ECOG PS (0)	0.728 (0.377–1.403)	0.343		
mALBI grade (1/2a)	0.585 (0.327–1.047)	0.071	0.760 (0.394–1.464)	0.412
BCLC stage (B)	0.855 (0.474–1.544)	0.604		
Portal vein invasion (+)	1.820 (0.977–3.390)	0.059	1.533 (0.794–2.962)	0.203
Extrahepatic metastasis (–)	1.096 (0.608–1.975)	0.760		
AFP (≥400 ng/mL)	1.619 (0.887–2.953)	0.116		
DCP (≥400 mAU/mL)	1.240 (0.636–2.418)	0.527		
Prior first-line ICI regimen (Atz/Bev)	1.472 (0.708–3.063)	0.301		
Disease control with prior ICI	0.740 (0.381–1.435)	0.373		

Abbreviations: PFS, progression-free survival; ECOG PS, Eastern Cooperative Oncology Group performance status; mALBI, modified albumin–bilirubin; BCLC, Barcelona Clinic Liver Cancer; AFP, alpha-fetoprotein; DCP, des-gamma-carboxy prothrombin; ICI, immune checkpoint inhibitor; HR, hazard ratio; CI, confidence interval.

**Table 6:** Univariate and multivariate analyses of prognostic factors for OS after initiation of second-line lenvatinib in the overall cohort.

	Univariate Analysis		Multivariate Analysis	
Factors	HR (95% CI)	*p* Value	HR (95% CI)	*p* Value
Age (≥75 years)	0.565 (0.282–1.130)	0.106		
Sex (female)	0.491 (0.188–1.280)	0.146		
ECOG PS (0)	0.357 (0.169–0.753)	0.007	0.294 (0.123–0.701)	0.006
mALBI grade (1/2a)	0.229 (0.109–0.429)	<0.001	0.169 (0.075–0.381)	<0.001
BCLC stage (B)	0.567 (0.280–1.147)	0.115		
Portal vein invasion (+)	1.671 (0.804–3.474)	0.169		
Extrahepatic metastasis (–)	0.547 (0.272–1.099)	0.090	0.767 (0.286–2.055)	0.598
AFP (≥400 ng/mL)	1.764 (0.881–3.531)	0.103		
DCP (≥400 mAU/mL)	1.733 (0.744–4.033)	0.202		
Prior first-line ICI regimen (Atz/Bev)	0.696 (0.308–1.571)	0.383		
Disease control with prior ICI	0.509 (0.241–1.078)	0.078	0.474 (0.176–1.279)	0.139

Abbreviations: OS, overall survival; ECOG PS, Eastern Cooperative Oncology Group performance status; mALBI, modified albumin–bilirubin; BCLC, Barcelona Clinic Liver Cancer; AFP, alpha-fetoprotein; DCP, des-gamma-carboxy prothrombin; ICI, immune checkpoint inhibitor; HR, hazard ratio; CI, confidence interval.

**Table 7:** Safety and treatment exposure of lenvatinib according to prior ICI regimen in the overall cohort.

Adverse Events, n (%) (Any Grade /≥Grade 3)	Overall Cohort (*n* = 56)	After Atz/Bev (*n* = 41)	After Dur/Tre (*n* = 15)
Appetite loss	28 (50.0)/3 (5.4)	20 (48.8)/1 (2.4)	8 (53.3)/2 (13.3)
General fatigue	26 (46.4)/4 (7.1)	18 (43.9)/2 (4.9)	8 (53.3)/2 (13.3)
Proteinuria	19 (33.9)/7 (12.5)	18 (43.9)/6 (14.6)	1 (6.7)/1 (6.7)
Hand–foot syndrome	15 (26.8)/0 (0.0)	12 (29.3)/0 (0.0)	3 (20.0)/0 (0.0)
Thyroid dysfunction	11 (19.6)/1 (1.8)	8 (19.5)/1 (2.4)	3 (20.0)/0 (0.0)
Diarrhea	10 (17.9)/2 (3.6)	8 (19.5)/2 (4.9)	2 (13.3)/0 (0.0)
Hypertension	9 (16.1)/1 (1.8)	5 (12.2)/1 (2.4)	4 (26.7)/0 (0.0)
Bleeding	3 (5.4)/0 (0.0)	2 (4.9)/0 (0.0)	1 (6.7)/0 (0.0)
Rash	3 (5.4)/0 (0.0)	3 (7.3)/0 (0.0)	0 (0.0)/0 (0.0)
Fever	3 (5.4)/0 (0.0)	3 (7.3)/0 (0.0)	0 (0.0)/0 (0.0)

Abbreviations: ICI, immune checkpoint inhibitor; Atz/Bev, atezolizumab plus bevacizumab; Dur/Tre, durvalumab plus tremelimumab; AE, adverse event. Adverse events were graded according to CTCAE version 5.0.

**Table 8:** Relative dose intensity of second-line lenvatinib during the first 6 weeks after treatment initiation.

	After Atz/Bev (*n* = 40)	After Dur/Tre (*n* = 14)	*p*-Value
**Overall cohort (*n* = 54)**			
RDI of lenvatinib, %; median (range)	50.0 (7.9–100)	40.0 (3.6–100)	0.170
**Child–Pugh class A subgroup (*n* = 41)**			
RDI of lenvatinib, %; median (range)	53.0 (22.2–100)	46.0 (10.6–100)	0.362

Abbreviations: RDI, relative dose intensity; Atz/Bev, atezolizumab plus bevacizumab; Dur/Tre, durvalumab plus tremelimumab. Patients who had not completed 6 weeks of follow-up at the time of analysis were excluded from the RDI analysis.

**Table 9:** Post-lenvatinib treatment after discontinuation of second-line lenvatinib according to prior ICI regimen.

**Overall Cohort**		
Post-lenvatinib treatment	**After Atz/Bev (*n* = 32)**	**After Dur/Tre (*n* = 7)**
Subsequent treatment	24 (75.0%)	3 (42.9%)
Systemic therapy	21 (65.6%)	3 (42.9%)
TACE/HAIC only	3 (9.4%)	0 (0.0%)
Best supportive care	8 (25.0%)	4 (57.1%)
**Child–Pugh class A subgroup**		
Post-lenvatinib treatment	**After Atz/Bev (*n* = 27)**	**After Dur/Tre (*n* = 3)**
Subsequent treatment	21 (77.8%)	2 (66.7%)
Systemic therapy	18 (66.6%)	2 (66.7%)
TACE/HAIC only	3 (11.1%)	0 (0.0%)
Best supportive care	6 (22.2%)	1 (33.3%)

Abbreviations: Atz/Bev, atezolizumab plus bevacizumab; Dur/Tre, durvalumab plus tremelimumab; TACE, transarterial chemoembolization; HAIC, hepatic arterial infusion chemotherapy. Patients who remained on lenvatinib treatment at the time of analysis were excluded from this analysis.

## Data Availability

The data presented in this study are available on request from the corresponding author. The data are not publicly available due to privacy and institutional restrictions.

## Abbreviations

The following abbreviations are used in this manuscript:

AFP	alpha-fetoprotein
AE	adverse event
Atz/Bev	atezolizumab plus bevacizumab
BCLC	Barcelona Clinic Liver Cancer
CI	confidence interval
CR	complete response
CTCAE	Common Terminology Criteria for Adverse Events
DCP	des-gamma-carboxy prothrombin
DCR	disease control rate
Dur/Tre	durvalumab plus tremelimumab
ECOG	Eastern Cooperative Oncology Group
FGFR	fibroblast growth factor receptor
HCC	hepatocellular carcinoma
HBV	hepatitis B virus
HCV	hepatitis C virus
HR	hazard ratio
ICI	immune checkpoint inhibitor
mALBI	modified albumin–bilirubin
mRECIST	modified Response Evaluation Criteria in Solid Tumors
NE	not evaluable
ORR	objective response rate
OS	overall survival
PD	progressive disease
PFS	progression-free survival
PR	partial response
RDI	relative dose intensity
RECIST	Response Evaluation Criteria in Solid Tumors
SD	stable disease
TKI	tyrosine kinase inhibitor
VEGF	vascular endothelial growth factor
VEGFR	vascular endothelial growth factor receptor
